# Long-term prospective assessment of quality of life and physical function in malignant lymphoma patients undergoing chemotherapy

**DOI:** 10.1097/MD.0000000000050246

**Published:** 2026-08-21

**Authors:** Ryuichi Kasahara, Takaaki Fujita, Ryohei Jinbo, Yuichi Yamamoto, Tatsuyuki Kai, Miki Furukawa, Hideo Kimura, Yasuyuki Taki, Jack B. Fu, Shinichiro Morishita

**Affiliations:** a Department of Smart-Aging Research Center, Tohoku University, Miyagi, Japan; b Department of Occupational Therapy, School of Health Sciences, Fukushima Medical University, Fukushima, Japan; c Department of Rehabilitation, Kita-Fukushima Medical Center, Fukushima, Japan; d Department of Hematology, Kita-Fukushima Medical Center, Fukushima, Japan; e Department of Palliative, Rehabilitation and Integrative Medicine, The University of Texas MD Anderson Cancer Center, Houston, TX; f Department of Physical Therapy, School of Health Sciences, Fukushima Medical University, Fukushima, Japan.

**Keywords:** health state utility values, health-related quality of life, long-term follow-up, malignant lymphoma, physical function

## Abstract

This study aims to evaluate longitudinal changes in health state utility values, health-related quality of life, physical function, nutritional status, and fatigue in patients with malignant lymphoma from the start of chemotherapy to 1 year post-chemotherapy and clarify the implications for survivorship care planning. This prospective observational study included 21 patients hospitalized for chemotherapy who also received exercise therapy. Assessments were conducted at baseline at admission, post-chemotherapy at discharge, as well as 6 months and 12 months after discharge (6M and 12M, respectively). Quality of life was assessed using the Short-Form 6-Dimension (SF-6D) and Medical Outcomes Study 36-Item Short-Form Health Survey (SF-36); physical function using handgrip strength, knee extension strength, and the 6-minute walk test; nutritional status using the Mini Nutrition Assessment^®^; and fatigue using the Brief Fatigue Inventory. Temporal changes were analyzed using repeated measures analysis of variance with Bonferroni correction. SF-6D scores improved significantly from 0.571 ± 0.082 at baseline at admission to 0.693 ± 0.096 at 12M (*P* < .01). Significant improvements were also found in most SF-36 domains over time, including physical functioning, role physical, general health, vitality, social functioning, role emotional, and mental health. Although no significant changes were observed in physical function, it remained stable throughout the study period. Mini Nutrition Assessment^®^ scores significantly improved at 6M (*P *< .05), and Brief Fatigue Inventory scores significantly improved at both 6M and 12M (*P* < .05 and *P* < .01, respectively). Chemotherapy patients with malignant lymphoma showed significant improvements in SF-6D, SF-36, and fatigue over the 1 year period following discharge, while physical function and nutritional status remained stable. These findings emphasize the need for ongoing rehabilitation and nutritional support during and after chemotherapy and provide evidence to inform long-term survivorship care strategies.

## 1. Background

Malignant lymphoma originates in the lymphatic system, and requires long-term, high-dose chemotherapy, which can significantly impact patients’ physical and mental functions, leading to muscle weakness, reduced endurance, and a decline in health-related quality of life (HRQOL).^[1–4]^ In particular, during and after chemotherapy, factors such as muscle weakness, fatigue, and poor nutritional status can limit daily activities, and may hinder social reintegration and long-term health maintenance.^[5–8]^ Therefore, preserving and improving physical function and HRQOL during chemotherapy have become increasingly important.

One method for improving physical function and HRQOL is exercise therapy. Systematic reviews and meta-analyses of exercise interventions in patients with malignant lymphoma have demonstrated their effectiveness in improving muscle strength, muscle mass, aerobic function, and HRQOL.^[8–10]^ However, most previous studies have focused on short-term outcomes, either during or immediately after chemotherapy, or on outcomes at specific time points following treatment.^[11–13]^ Therefore, the long-term course of physical function and HRQOL in patients with malignant lymphoma remains poorly understood.

Furthermore, the temporal changes in health state utility values (HSUVs) in patients with malignant lymphoma remain unclear. HSUVs are important indicators from a health economics perspective, and are widely used to evaluate the effectiveness and cost-effectiveness of medical interventions for cancer patients.^[14,15]^ Therefore, clarifying the long-term course of HSUVs using the Short-Form 6-Dimension (SF-6D) may contribute both to the development of optimal care plans tailored to individual patient needs and to the appropriate allocation of medical resources.

The purpose of the present study was to clarify the temporal changes in HSUVs, HRQOL, physical function, nutritional status, and fatigue in patients with malignant lymphoma from before the start of chemotherapy to 1 year after its completion. The comprehensive and longitudinal evaluation of these indicators is expected to contribute to the improvement of rehabilitation programs and supportive care from before chemotherapy initiation through the post-chemotherapy follow-up period.

## 2. Methods

**Figure 1.**
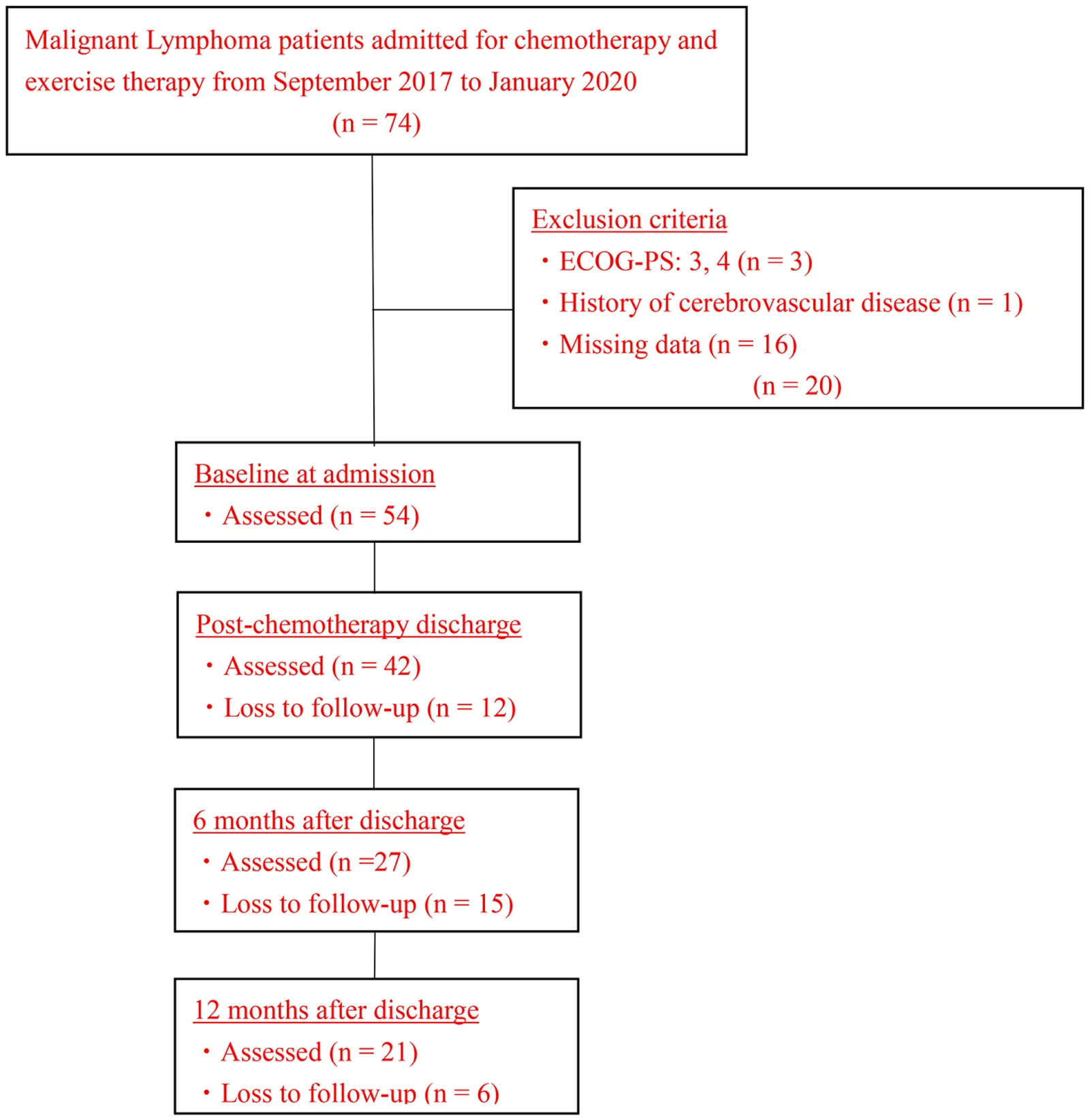
Study flow chart. Flow diagram showing patient enrollment, exclusion, and follow-up during the study period.

This study is a prospective observational study. The study population included patients who were hospitalized and received both chemotherapy and exercise therapy between September 2017 and January 2020. Exclusion criteria included an Eastern Cooperative Oncology Group Performance Status (ECOG-PS) of ≥3 at admission; the presence of musculoskeletal or cerebrovascular disorders that could interfere with physical function assessment; complications that hinder physical function assessment; mental disorders or other conditions preventing completion of the questionnaire; missing data required for analysis; judgment by the attending physician deeming inclusion inappropriate; and lack of informed consent for study participation. This study was designed as an exploratory prospective observational study; therefore, a formal a priori sample size calculation was not performed and a convenience sampling was used. All eligible patients who met the inclusion criteria during the study period were included (Fig. 1). All chemotherapy and physical therapy were administered during hospitalization.

This study was performed in line with the principles of the Declaration of Helsinki. Approval was granted by the Ethics Committee of the Kita Fukushima Medical Center (Approval No. 74-3). Written informed consent was obtained from all participants.

## 3. Assessments and measurements

### 3.1. Assessments

Basic information, including age, sex, weight, body mass index, ECOG-PS at admission, type of malignant lymphoma, type of chemotherapy, exercise therapy implementation rate, and length of hospital stay, was collected from medical records.

### 3.2. Measurements

HRQOL was assessed using the Medical Outcomes Study 36-Item Short-Form Health Survey (SF-36).^[16]^ This comprehensive instrument evaluates 8 subscales: physical functioning (PF), role physical, bodily pain (BP), general health, vitality (VT), social functioning, role emotional, and mental health. Each subscale is scored on a scale of 0 to 100, with higher scores indicating better quality of life. HSUVs were calculated using the SF-6D derived from the SF-36.^[17]^ The SF-6D is a profile-based measure used to capture the effects of medical interventions. In comparison with other utility measures, The SF-6D comprises multiple items that cover 6 dimensions (PF, role limitation, social functioning, BP, MH, and VT),^[17,18]^ and is characterized by a minimal ceiling effect.^[19]^ Additionally, the SF-6D has been reported to demonstrate high sensitivity to changes in HRQOL due to cancer.^[20,21]^

Physical function assessment included measurements of grip strength, knee extension strength, and the 6-minute walk test (6MWT). Grip strength was measured twice on each side using a grip dynamometer (GRIP D; Takei Scientific Instruments Co., Ltd., Niigata, Japan), and the maximum value for each side was recorded. Knee extension strength was measured using a handheld dynamometer (μ-tas F1; ANIMA Co., Ltd., Tokyo, Japan) while the participant was seated at the edge of the bed with their knee flexed at 90°. A fixation belt was used to ensure measurement consistency. Three trials were conducted per side, and the maximum value for each side was recorded. For both grip and knee extension strength, the average of the maximum values from the right and left sides was used for analysis. The 6MWT was conducted in accordance with the standards set by the American Thoracic Society.^[22]^

Nutritional assessment was performed using the Mini Nutrition Assessment^®^ (MNA^®^).^[23]^ The MNA^®^ is a comprehensive nutritional assessment tool originally developed for the elderly, consisting of 18 questions on dietary intake, weight change, physical function, mental stress, anthropometric data, medication, and health perception, and is also used to assess nutrition in patients with hematological cancer.^[24,25]^ The total MNA^®^ score ranges from 0 to 30, with higher scores indicating better nutritional status.

Fatigue was assessed using the Brief Fatigue Inventory (BFI),^[26]^ a subjective measure of fatigue in cancer patients. The BFI consists of 9 questions, each scored on a scale of 0 to 10, with an average score calculated. A higher average score indicates more severe fatigue.

Measurements were conducted at the following time points: baseline at admission (BL), post-chemotherapy discharge (PC), 6 months after discharge (6M), and 12 months after discharge (12M).

### 3.3. Analysis

Descriptive statistics were performed for the basic characteristics of all participants. Temporal changes in HSUVs, average grip strength, average knee extension strength, 6MWT, MNA^®^, and BFI at each time point (BL, PC, 6M, and 12M) were analyzed using repeated measures one-way analysis of variance with Bonferroni multiple comparisons. Statistical analyses were performed using SPSS Statistics version 29 (SPSS Japan Inc., Tokyo, Japan). A *P*-value of <.05 was considered statistically significant.

## 4. Result

A total of 21 patients were evaluated from before the start of chemotherapy until 1 year after its completion. The baseline characteristics of the patients were as follows: mean age 66.9 ± 9.5 years; height 156.5 ± 7.4 cm; weight 55.3 ± 11.1 kg; and 38.1% were male. The types of malignant lymphoma were Diffuse large B-cell lymphoma (71.4%), Follicular Lymphoma (19.0%), Mucosa-associated lymphoid tissue lymphoma (4.8%), peripheral T-cell lymphoma (4.8%). The ECOG-PS was 0 in 76.2% of cases. The mean hospitalization period was 172.1 ± 37.4 days, and the implementation rate of exercise therapy was 95.9% (Table 1).

**Table 1:** Clinical and demographic characteristics data of patients with malignant lymphoma.

Characteristics	Overall n = 21
Age, yr	66.9 ± 9.5
Height, cm	156.5 ± 7.4
Body weight, kg	55.3 ± 11.1
Body mass index, kg/m^2^	22.5 ± 3.9
Sex	
Male	8 (38.1%)
Female	13 (61.9%)
Malignant lymphoma type	
DLBCL	15 (71.4%)
FL	4 (19.0%)
MALT	1 (4.8%)
PTCL	1 (4.8%)
ECOG-PS	
0	16 (76.2%)
1	5 (23.8%)
Length of hospitalization, d	172.1 ± 37.4
Adherence to exercise therapy, %	95.9 ± 3.3

Mean ± standard deviations or number.

DLBCL = diffuse large B-cell lymphoma, ECOG-PS = Eastern Cooperative Oncology Group Performance Status, FL = follicular lymphoma, MALT = mucosa-associated lymphoid tissue lymphoma, PTCL = peripheral T-cell lymphoma.

The results of one-way analysis of variance and multiple comparison tests revealed significant differences in SF-6D scores between BL and 6M (*P* = .02), between BL and 12M (*P* < .01), and between PC and 12M (*P* = .04). Significant improvements were also observed in the following SF-36 subscales: PF between BL and 6M (*P* = .04) and between PC and 12M (*P* < .01); role physical between BL and 12M (*P* = .03) and between PC and 12M (*P* = .02); general health between BL and 12M (*P* = .02); VT between BL and PC, between BL and 6M, and between BL and 12M (all *P* < .01); social functioning between BL and 12M (*P* = .04) and between PC and 12M (*P* = .04); role emotional between BL and 12M (*P* < .01); MH between BL and PC, between BL and 6M, and between BL and 12M (all *P* < .01) (Table 2).

**Table 2:** Temporal changes in SF-6D and SF-36 of patients with malignant lymphoma.

Variables	BL	PC	6M	12M	*P*-value
SF-6D, mean	0.571 ± 0.082	0.619 ± 0.077	0.651 ± 0.090	0.693 ± 0.096	BL < 6M*, BL < 12M**, PC < 12M*
SF-36, points					
Physical functioning	67.1 ± 23.9	81.7 ± 15.0	82.1 ± 17.9	86.9 ± 11.9	BL < 6M*, BL < 12M**
Role physical	59.2 ± 31.4	58.3 ± 37.2	71.4 ± 28.1	86.0 ± 17.6	BL < 12M*, PC < 12M*
Bodily pain	66.2 ± 22.8	75.3 ± 25.6	69.3 ± 25.1	76.9 ± 24.0	
General Health	43.9 ± 15.4	55.1 ± 14.2	53.7 ± 12.6	58.1 ± 17.5	BL < 12M*
Vitality	42.3 ± 15.4	61.9 ± 16.8	61.0 ± 21.9	69.6 ± 19.4	BL < PC**, BL < 6M**, BL < 12M**
Social functioning	72.6 ± 31.5	72.6 ± 26.7	83.9 ± 19.8	92.9 ± 10.1	BL < 12M*, PC < 12M*
Role emotional	60.7 ± 27.3	75.0 ± 36.4	77.0 ± 21.7	92.5 ± 13.2	BL < 12M**
Mental health	54.0 ± 14.7	79.3 ± 15.1	77.4 ± 17.1	81.4 ± 13.2	BL < PC**, BL < 6M**, BL < 12M**

Mean ± standard deviations.

12M = 12 months after discharge, 6M = 6 months after discharge, BL = baseline at admission, PC = post-chemotherapy discharge, SF-36 = Medical Outcomes Study 36-Item Short-Form Health Survey, SF-6D = Short-Form 6-Dimension.

* *P* < 0.05.

** *P* < 0.01.

No significant changes were observed in physical function, including grip strength, knee extension strength, and 6MWT. The MNA^®^ showed significant improvement between BL and 6M (*P* = .02). The BFI showed significant differences between BL and 6M (*P* = .04) and between BL and 12M (*P* < .01) (Table 3).

**Table 3:** Temporal changes in physical function, nutritional status, and fatigue of patients with malignant lymphoma.

Variables	Baseline	PC	6M	12M	*P*-value
Average grip strength, kgf	21.6 ± 8.4	20.7 ± 7.3	22.6 ± 7.4	22.6 ± 8.3	
Average knee extension strength, kgf	22.4 ± 8.1	24.1 ± 8.5	26.9 ± 7.4	27.0 ± 7.7	
6-min walk test, m	443.1 ± 92.0	467.3 ± 82.5	490.6 ± 69.4	505.9 ± 80.9	
Mini nutritional assessment®, points	19.9 ± 3.8	22.8 ± 3.4	25.1 ± 2.7	22.2 ± 9.6	BL < 6M*
Brief fatigue inventory, points	3.20 ± 2.16	1.94 ± 2.03	1.62 ± 1.32	1.12 ± 1.69	BL < 6M*, BL < 12M**

Mean ± standard deviations.

12M = 12 months after discharge, 6M = 6 months after discharge, BL = baseline at admission, PC = post-chemotherapy discharge.

* *P* < 0.05.

** *P* < 0.01.

## 5. Discussion

This study comprehensively and longitudinally investigated SF-6D, SF-36, physical function, nutritional status, and fatigue in patients with malignant lymphoma, a condition previously poorly understood, from before the start of chemotherapy through 1 year after discharge. The novelty of this study lies in its demonstration of the long-term course of patients with malignant lymphoma, and the results provide important data to inform strategies for long-term patient support.

The findings demonstrated significant improvements in multiple items of SF-6D and SF-36 from before chemotherapy initiation to 1 year after its completion. Specifically, the average SF-6D score increased from 0.571 ± 0.082 pre-chemotherapy to 0.693 ± 0.096 1 year post-discharge. These findings align with previous studies reporting an increase in HSUVs before and after initial chemotherapy.^[27]^ In addition, it has been reported that the HRQOL in patients with malignant lymphoma is lowest at the start of chemotherapy and improves thereafter,^[28]^ which is consistent with the findings of the present study. In a previous SF-6D study involving patients with malignant lymphoma, Li et al^[29]^ reported an average SF-6D score of 0.714 ± 0.191 (mean age 48.4 years) in patients who achieved complete remission after initial chemotherapy. Additionally, Hays et al^[20]^ reported an initial SF-6D score of 0.718 in elderly patients (≥65 years) newly diagnosed with non-Hodgkin lymphoma, based on the Surveillance, Epidemiology, and End Results national cancer registry system and the Medicare Health Outcomes Survey, although the timing relative to chemotherapy was not specified. Furthermore, Kent et al^[30]^ reported an SF-6D score of 0.68 (mean age 69.9 years) for survivors who had been diagnosed an average of 75.9 months prior. The SF-6D scores of the patients in the present study were slightly low at 0.571 ± 0.082 before chemotherapy, but improved to 0.693 ± 0.096 1 year after discharge. Considering that the average age of the patients was relatively high at 66.9 years, this result is plausible. This study revealed a new finding that SF-6D scores in patients with malignant lymphoma did not decline but instead improved over the course of 1 year after discharge.

Regarding SF-36, previous research has shown that HRQOL in non-Hodgkin lymphoma survivors improves more than 2 years after diagnosis, potentially returning to levels comparable to those of the general population,^[31]^ The findings of the present study are consistent with this trajectory. Additionally, fatigue has been reported to be associated with HRQOL in multiple studies.^[32–36]^ In the present study, the BFI, an indicator of fatigue, showed significant improvement at 6 and 12M compared with the baseline at hospitalization. The parallel trends observed in SF-6D and fatigue scores suggest an important association between the 2 indicators. However, among the SF-36 subscales, only bodily pain did not show significant improvement. This finding is consistent with those of previous studies,^[12,37]^ and underscores the need for continued assessment and care regarding pain following chemotherapy.

Although no significant changes in physical function were observed, the finding that it remained stable without decline after discharge is an important discovery. On the other hand, the SF-36 PF scores showed a significant improvement after discharge compared with admission. The discrepancy between the lack of change in objective measures and the improvement in subjective PF scores may be attributed to reduced fatigue and enhanced physical self-efficacy. Supporting this interpretation, the BFI scores in the present study showed a significant improvement over time, suggesting that reduced fatigue may have positively influenced patients’ perceptions of their physical function. In cancer patients, reduced fatigue has been reported to be associated with improved HRQOL and increased physical activity.^[35,36]^

Regarding nutritional status, the MNA^®^ score improved from admission to discharge and again at 6 months post-discharge, with a significant improvement observed at 6 months compared with admission, and the status was generally maintained at 1 year post-discharge. The overall trend in nutritional status was stable, with no significant decline observed. However, given that previous studies have shown that nutritional status influences prognosis and survival in patients with malignant lymphoma,^[38–40]^ ongoing support following discharge is considered essential.

This study demonstrates the necessity of comprehensive support during and after chemotherapy, and by clarifying the temporal changes in HSUVs, it may provide foundational data for future health policy and clinical intervention studies. However, this study has several limitations. First, the sample size was relatively small, which may limit the statistical power and generalizability of the findings. In addition, patients with an ECOG-PS of ≥3 were excluded, which may have resulted in a relatively healthier cohort. Therefore, the degree of HRQOL recovery observed in this study may have been overestimated compared with the broader population of patients undergoing chemotherapy. Second, this was a single-center study, and therefore the results should be interpreted with caution when generalizing them to other clinical settings. Third, all participants received exercise therapy during hospitalization, which may have influenced the observed outcomes. Fourth, the possibility of survivorship bias should be considered, as patients who experienced severe clinical deterioration or recurrence may have been less likely to complete long-term follow-up. Finally, the absence of a control group without exercise therapy limits the ability to determine the independent effects of rehabilitation. Additionally, since the observation period is limited to 1 year, the long-term outcomes beyond this period remain unclear. Further research is warranted, including multicenter collaborative studies and larger-scale cohort studies with long-term follow-up.

## 6. Conclusions

This prospective observational study demonstrated that HSUVs, HRQOL, and fatigue significantly improved in patients with malignant lymphoma from the initiation of chemotherapy through 1 year post-discharge, while physical function and nutritional status remained stable. These findings highlight the importance of comprehensive and continuous supportive care during and after chemotherapy. The importance of exercise therapy^[41,42]^ and nutritional^[42,43]^ guidance for cancer survivors has also been highlighted in existing guidelines, and the findings of the present study are consistent with these recommendations. Moreover, the observed improvements in SF-6D scores provide valuable data for informing future health policy and clinical interventions aimed at optimizing long-term survivorship care in patients with malignant lymphoma. Further large-scale, multicenter studies with extended follow-up are warranted to validate and expand upon these findings.

## Author contributions

**Conceptualization:** Ryuichi Kasahara.

**Data curation:** Ryuichi Kasahara, Ryohei Jinbo, Yuichi Yamamoto.

**Formal analysis:** Ryuichi Kasahara, Takaaki Fujita, Shinichiro Morishita.

**Funding acquisition:** Ryuichi Kasahara, Shinichiro Morishita.

**Methodology:** Ryuichi Kasahara, Takaaki Fujita, Ryohei Jinbo, Tatsuyuki Kai, Shinichiro Morishita.

**Project administration:** Ryuichi Kasahara.

**Resources:** Ryuichi Kasahara, Ryohei Jinbo, Yuichi Yamamoto.

**Software:** Ryuichi Kasahara.

**Visualization:** Ryuichi Kasahara.

**Supervision:** Takaaki Fujita, Tatsuyuki Kai, Yasuyuki Taki, Jack B Fu, Shinichiro Morishita.

**Validation:** Takaaki Fujita, Ryohei Jinbo, Yuichi Yamamoto, Shinichiro Morishita.

**Writing – original draft:** Ryuichi Kasahara.

**Writing – review & editing:** Ryuichi Kasahara, Takaaki Fujita, Ryohei Jinbo, Yuichi Yamamoto, Tatsuyuki Kai, Miki Furukawa, Hideo Kimura, Yasuyuki Taki, Jack B Fu, Shinichiro Morishita.
